# Fioreze C, Dias RG, Dóri LV, Bregolin L, Comin MD. Sintomas de ansiedade e depressão em adolescentes: estudo transversal, Passo Fundo, 2024. Epidemiol Serv Saude. 2026:35;e20250901.

**DOI:** 10.1590/S2237-96222026v35e20250901.b

**Published:** 2026-04-27

**Authors:** Pablo Guilherme Caldarelli

**Affiliations:** 1 Universidade Estadual de Londrina, Londrina, PR, Brazil Londrina PR Brazil

## Peer review B

## Questionnaire

Does the manuscript contain new and significant information to justify publication? Yes

Does the Abstract (Summary) clearly and accurately describe the content of the article? Yes

Is the problem significant and concisely stated? Yes

Are the methods described comprehensively? Yes

Are the interpretations and conclusions justified by the results? Yes

Is adequate reference made to other work in the field? Yes

## Manuscript Structure

Length of article is: Adequate

Number of tables is: Adequate

Number of figures is: Adequate

Please state any conflict(s) of interest that you have in relation to the review of this paper. None

Interest: Good

Quality: Good

Originality: Average

Overall: Good

Recommendation: Minor Revision

## Comments

Prezados editores e autores,

Encaminho minhas considerações e sugestões referentes à avaliação do manuscrito: RESS-2025-0901 - Prevalência e Fatores Associados à Depressão e Ansiedade em Adolescentes: um Estudo Transversal de Base Escolar

O manuscrito encontra-se coerente com o foco e escopo da Revista Epidemiologia e Serviços de Saúde - RESS. O artigo encontra-se relacionado a um tema original e de relevância para a área de Saúde Mental/ Saúde Pública.

Encaminho as recomendações abaixo de forma hierarquizada, separando-as em itens críticos, importantes e desejáveis para melhor direcionamento das alterações dos autores.

APONTAMENTOS CRÍTICOS

A INTRODUÇÃO está coerente com o conteúdo do manuscrito. Os autores apresentam o problema de pesquisa, contudo, poderiam apresentar uma melhor abordagem sobre a contribuição do manuscrito e da temática para o Sistema Único de Saúde (SUS) e o cuidado integral na Rede de Atenção Psicossocial (RAPS).

Os MÉTODOS foram executados de maneira suficiente para produzir resultados confiáveis. O delineamento escolhido é adequado para responder à questão de pesquisa (estudo transversal). Contudo, há necessidade de maiores detalhes na descrição da coleta de dados (p.05). Os autores poderiam trazer uma abordagem descritiva das escolas participantes do estudo.

Os autores devem revisar a descrição da análise estatística, esclarecendo o modelo utilizado para estimar as razões de prevalência.

APONTAMENTOS IMPORTANTES

O RESUMO encontra-se estruturado e com escrita satisfatória, apresenta com clareza os principais resultados da pesquisa, com números que apoiam as afirmações, e seus métodos esclarecem como tais resultados foram obtidos. Os autores devem incluir o local do estudo no resumo (p.03 linha14).

Na DISCUSSÃO, há necessidade de explorar melhor e incluir reflexão mais aprofundada sobre como os resultados podem ser generalizados e aplicados em outros contextos, justificando a relevância do estudo para diferentes populações.

Os autores poderiam reforçar a discussão sobre implicações práticas para políticas públicas, programas e projetos no fortalecimento de redes de apoio no ambiente escolar e comunitário (p.09 linha 46), com destaque para ações interprofissionais e intersetoriais no contexto do SUS.

APONTAMENTOS DESEJÁVEIS

O TÍTULO está de acordo com o conteúdo do manuscrito. Contudo, sugiro aos autores a inserção do cenário do estudo no título - município de grande porte do Rio Grande do Sul (p.03 linha 07-08).

Os autores poderiam aprofundar a discussão sobre a ausência da rede de apoio social como preditor significativo no Chisquared Automatic Interaction Detection (CHAID), considerando possíveis hipóteses para esse resultado.

Sugiro aos autores revisar toda formatação e realizar correções relacionadas à língua portuguesa (ortografia e gramática) em todo o corpo do texto.

Diante do exposto, considerando a atual apresentação do manuscrito, minha recomendação é: CORREÇÕES MENORES.

